# Potential role of the ocular surface microbiome in dry eye: microbial interactions and symptom alleviation

**DOI:** 10.1128/msystems.00104-26

**Published:** 2026-03-23

**Authors:** Joon-Young Park, Chang Ki Yoon, Jin-Jae Lee, Young Joo Shin, Bong-Soo Kim

**Affiliations:** 1 Department of Life Science, Multidisciplinary Genome Institute, Hallym University 26727, Chuncheon, Gangwon-do, Republic of Korea; 2 Department of Ophthalmology, Seoul National University Hospital 58927, Seoul, Republic of Korea; 3 Department of Ophthalmology, Hallym University Medical Center, Hallym University College of Medicine 96664, Seoul, Republic of Korea; 4 Hallym BioEyeTech Research Center, Hallym University College of Medicine 96664, Seoul, Republic of Korea; 5 Department of Nutritional Science and Food Management, Ewha Womans University 26717, Seoul, Republic of Korea; 6 Global Food and Nutrition Research Institute, Ewha Womans University 26717, Seoul, Republic of Korea; University of Southampton, Southampton, United Kingdom

**Keywords:** ocular surface microbiome, dry eye, meibomian gland dysfunction, microbiome modulation

## Abstract

Dry eye is a prevalent ocular disorder characterized by tear film instability, inflammation, and ocular discomfort. Although the ocular surface (OS) microbiome contributes to immune regulation and pathogen defense, its role in dry eye pathophysiology remains unclear. Therefore, the present study aimed to characterize alterations in the OS microbiome of patients with dry eye undergoing cyclosporin A or NewHyalUni treatment and to identify their potential roles related to clinical improvement. Patients with dry eye were treated with either cyclosporin A and NewHyalUni drop combination or NewHyalUni alone. OS samples were collected before and after treatment, and the microbiome was analyzed by whole metagenome sequencing. Potential contaminants were removed before downstream analysis to account for the low-biomass nature of OS samples. Clinical evaluations included symptom scores and the assessment of meibomian gland dysfunction (MGD). No significant differences in the overall microbial composition were observed between the treatment groups. Nevertheless, both groups demonstrated symptomatic improvement. OS microbiome alterations were strongly correlated with improvements in MGD scores. Moreover, microbial interactions were found to shift following treatment. Key species (*Staphylococcus epidermidis*, *Staphylococcus pseudintermedius*, *Streptomyces lividans,* and *Edwardsiella tarda*) were identified as potential mediators of MGD score improvement by modulating microbiome functions and suppressing inflammation-associated species. Although distinct treatment regimens did not lead to divergent microbiome profiles, symptomatic improvement was associated with alterations in a specific microbiome. These findings highlight the OS microbiome’s potential role in dry eye and support the development of microbiome-based therapeutic strategies.

**IMPORTANCE:**

Dry eye is a common ocular disorder with complex pathophysiology that extends beyond tear deficiency and inflammation. Despite growing evidence of host-microbiome interactions at mucosal surfaces, the contribution of the ocular surface (OS) microbiome to dry eye remains poorly understood. Our findings in this study reveal that shifts in specific taxa and ecological interactions correlate with improvements in meibomian gland function and dry eye symptoms, even in the absence of major changes in overall microbiota. By identifying microbial signatures potentially linked to clinical improvement, we provide systems-level insight into the role of low-biomass microbiomes in ocular health. This work expands the current understanding of microbiome-host dynamics in non-gut environments and supports future development of microbiome-informed therapeutic strategies.

**CLINICAL TRIALS:**

This study is registered with ClinicalTrials.gov as NCT06936462.

## INTRODUCTION

The ocular surface (OS) is constantly exposed to environmental factors (e.g., microbes and microparticles) that can cause infectious, toxic, or allergenic reactions. Various protective mechanisms, comprising mechanical (blinking and tear secretion), chemical (lysozymes, lactoferrins, and defensins), and immunological (neutrophils, secretory IgA, and lymphocytes) defenses, protect against potential pathogens (1–3). These protective mechanisms help reduce microbial colonization of the OS (4). Early culture-dependent studies have recognized the presence of microbes on the OS (5). Recently, culture-independent sequencing methods have provided a more comprehensive characterization of the OS microbiome (6–10). Similar to other human microbiomes, the OS microbiome plays key roles in immune regulation (11, 12) and pathogen defense (13). Accordingly, previous studies have implicated OS microbiome dysbiosis in various ocular diseases, including conjunctivitis, keratitis, endophthalmitis, blepharitis, and dry eye disease (14–16).

Dry eye is a prevalent ocular condition that results from decreased tear production or increased tear evaporation, which results in tear film destabilization, leading to OS inflammation and ocular discomfort (17, 18). Dry eye syndrome is a chronic condition that often requires long-term management to alleviate symptoms and prevent further damage to the OS. Although previous studies have reported differences in the OS microbiome between patients with dry eye and controls (19–21), there are inconsistent findings across studies. Several studies have observed that patients with dry eye have a lower microbiome diversity than those without dry eye (8, 16, 19). However, taxonomic differences between patients with dry eye and controls have not been uniform. For example, in Chinese participants with dry eye, the relative abundance of Bacteroidota (formerly Bacteroidetes) was higher, whereas those of *Pseudomonas* and Pseudomonadota (formerly Proteobacteria) were lower (19). Another study on Chinese participants reported an increased abundance of Actinomycetota (formerly Actinobacteria) and decreased levels of *Cyanobacteria* and *Bacteroides* in dry eye patients with Sjögren’s syndrome compared with controls (16). However, no significant compositional differences were observed between the Sjögren’s syndrome group and the non-dry eye group in studies conducted in Korea and Texas (8, 22).

These discrepancies may be attributed to individual variations, differences in sampling methods, and the OS microbiome’s inherently low biomass (10, 23, 24). Considering that the OS has a low bacterial load, 16S rRNA gene amplification is commonly used for microbiota analysis. However, because this method has a high sensitivity, the risk of detecting clinically irrelevant bacteria or contaminants introduced during experimental procedures is high (23, 25). Furthermore, a previous study has observed variations in the microbiota composition depending on the sampling technique, including tear-impregnated paper and conjunctival swabs (10). The inconsistency in taxonomic composition across studies suggests the presence of individual-specific core microbiomes, instead of a universally recognized core OS microbiome (24, 26). Nevertheless, several studies have highlighted the role of the OS microbiome in maintaining OS homeostasis by modulating host immune responses and influencing susceptibility to infection (11, 27–29). Obtaining knowledge of the OS microbiome is crucial for developing microbiome-based treatment and management strategies for ocular disorders.

The standard treatments for dry eye syndrome and OS disease typically include artificial tears, anti-inflammatory agents, and medications that enhance tear secretion. Among them, topical cyclosporine A (CsA), an immunomodulatory drug, has been approved for the treatment of dry eye syndrome. CsA suppresses immune activation and reduces OS inflammation, playing an important role in the development and progression of dry eye syndrome. CsA can help alleviate symptoms and improve tear film stability by targeting this underlying inflammation. In patients with dry eye, topical CsA has been shown to promote tear and mucus secretion while providing symptomatic relief. However, despite its therapeutic benefits, the potential impact of CsA on the OS microbiome remains unclear.

A longitudinal study of the OS microbiome in patients receiving treatment may reveal significant information after reducing possible contaminants. However, few studies have examined the shifts in and the potential functions of the OS microbiome during dry eye treatment. Moreover, the differential effect of therapeutic eye drops on the OS microbiome remains to be fully elucidated. In the present study, the longitudinal changes in the OS microbiome of patients with dry eye were analyzed during CsA and NewHyalUni treatment using whole metagenome sequencing (WMS), following the removal of potential contaminants. Furthermore, the potential role of the OS microbiome in modulating inflammatory responses was assessed. The findings of this study contribute to a deeper understanding of the OS microbiome in dry eye disease.

## MATERIALS AND METHODS

### Study design and sample collection

This study is a randomized, double-blind, and prospective trial. The inclusion criteria were as follows: patients aged 19 years or older with symptoms of dry eye syndrome. Fifty participants were randomly assigned to either the CsA group (CsA twice daily and hyaluronic acid twice daily, *n* = 25) or the control group (hyaluronic acid four times daily, *n* = 25). This study used 0.05% CsA (Cyporin N Eye Drops, Taejoon Pharm, Republic of Korea) and 0.15% hyaluronic acid (NewHyalUni Eye Drops, Taejoon Pharm). The examiner and sample collector were separated to maintain blinding.

The exclusion criteria were as follows: use of topical ocular drops or systemic medications (e.g., systemic steroids, immunomodulators, and tetracyclines) that could influence the study outcomes and presence of conjunctivitis, anterior blepharitis, *Demodex* infestation, parasitic eye infections, unresolved ocular trauma, OS healing disorders, history of penetrating keratoplasty, use of contact lens, or any ophthalmic surgical procedure within 3 months before baseline. In addition, participants with a single functional eye, pregnant or breastfeeding women, and participants at risk of pregnancy without contraception were excluded.

**Fig 1:**
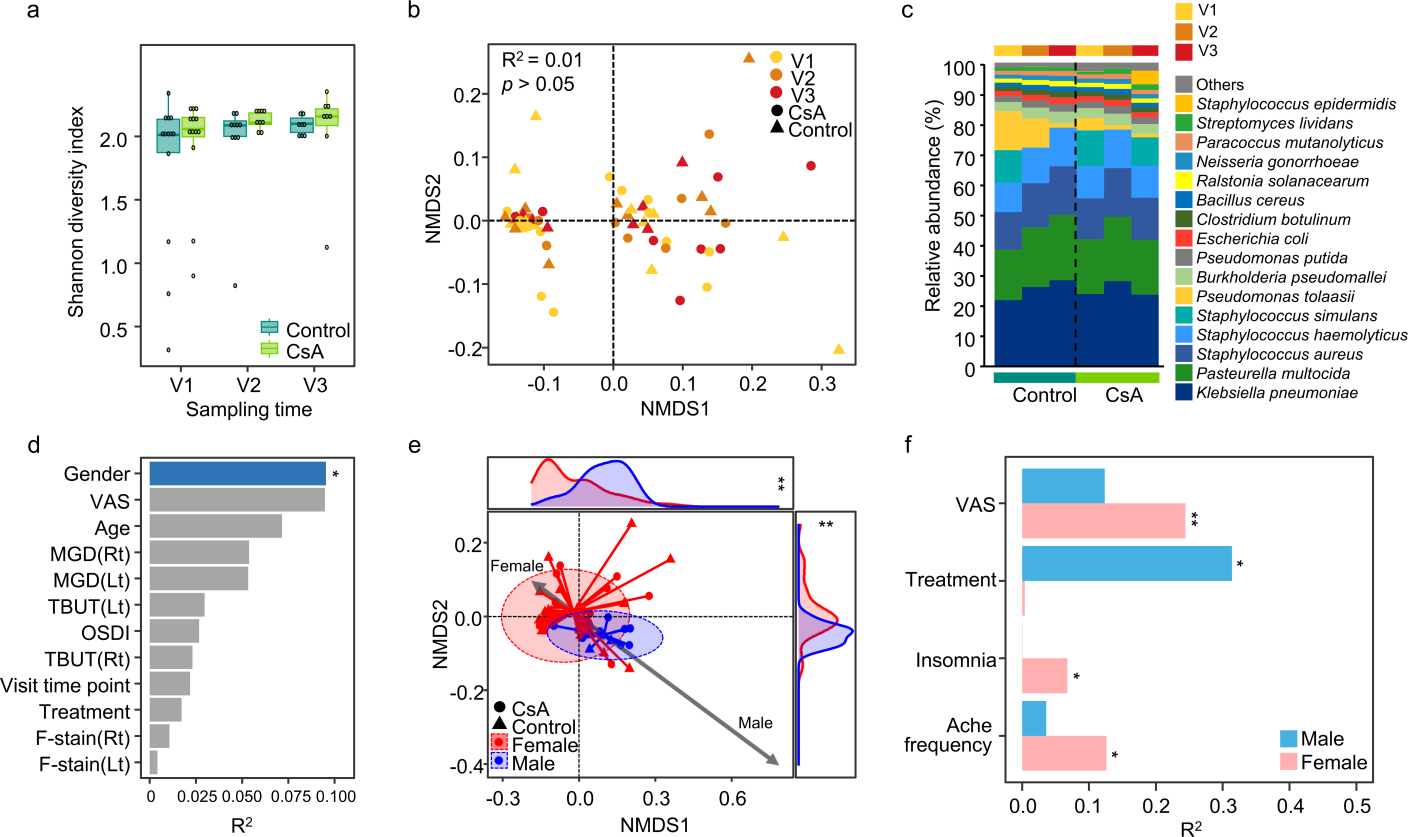
Comparison of microbiota across samples and identification of confounding factors. (**a**) The microbial diversity was compared across samples at different time points. The statistical significance was calculated using Dunn’s test with Benjamini–Hochberg false discovery rate correction. (**b**) The microbial composition was visualized using a nonmetric multidimensional scaling (NMDS) plot to compare treatment groups across time points. *P*-values were calculated using permutational multivariate analysis of variance. (**c**) The dominant species in each sample group (defined as >1% of relative abundance in each group) were compared using bar plots. (**d**) The host factors and clinical scores that were significantly associated with variations in the OS microbiota were identified using the EnvFit model. A significant factor is highlighted in blue. (**e**) The differences in OS microbiota between female and male subjects were visualized using NMDS plots. The NMDS1 and NMDS2 axis scores were compared between groups using the Wilcoxon rank-sum test. (**f**) The covariates that were significantly associated with OS microbiota variation were determined using the EnvFit model. VAS, visual analog scale; MGD, meibomian gland dysfunction; TBUT, tear break-up time; OSDI, ocular surface disease index; F-stain, fluorescein eye stain. **P < 0.01, *P < 0.05.

Clinical data and OS samples were collected at baseline before eye drop administration (V1) and at 4 (V2) and 12 weeks (V3) after treatment initiation (Fig. 1a). Out of the 50 participants at V1, 21 participants from each group completed follow-up at V2. Moreover, 14 participants in the CsA group and 18 participants in the control group remained at V3. The OS samples for microbiome analysis were obtained from the inferior conjunctival sac using a sterile mixed cellulose ester membrane (Millipore, Merck). The OS samples were collected from 13 participants in the CsA and control groups, respectively. All OS samples were stored at −80°C until DNA extraction for microbiome analysis. Because OS specimens are inherently low biomass, not all collected samples yielded sufficient DNA or sequencing quality after stringent decontamination and quality filtering. Therefore, WMS analyses were performed in a balanced subset with longitudinal OS samples and quality-filtered data (*n* = 13 per group) to enable within-subject comparisons across visits.

### Collection of clinical data

The symptoms were assessed using the ocular surface disease index (OSDI), visual analog scale (VAS) for pain, and the modified Standard Patient Evaluation of Eye Dryness questionnaire. Meanwhile, the tear film stability was evaluated by measuring the tear break-up time (TBUT). Fluorescein staining of the cornea was performed by applying a drop of sterile saline to a sterile fluorescein strip. The time between a normal blink and the first appearance of a dry spot in the tear film was recorded after the participant blinked naturally three times. The average of three repeated measurements was used for the analysis.

OS inflammation was assessed using the fluorescein staining score, which was graded according to the Oxford scoring system (30). The severity of meibomian gland and eyelid inflammation and lid margin hyperemia was graded as none, mild, moderate, or severe. Tear secretion was measured using the Schirmer strip without anesthesia. The Schirmer strip was placed at the temporal third of the lower eyelid, between the lower palpebral conjunctiva and the lower bulbar conjunctiva. After 5 min, the length of the tear fluid absorbed by the strip was measured in millimeters.

### DNA extraction and whole metagenome sequencing

Total DNA was extracted from the collected OS samples using the RNeasy PowerMicrobiome Kit (Qiagen, Inc., Valencia, CA, USA). The DNA concentration was measured with the BioPhotometer D30 using a μCuvette G1.0 (Eppendorf, Hamburg, Germany). Twelve negative controls, comprising sampling membranes, DNA-free water added to the DNA extraction kit, and DNA-free water added to the sequencing library preparation kit, were included to eliminate potential contaminants during the experimental process. These negative controls were sequenced alongside the 61 OS samples.

For whole metagenome sequencing, the extracted DNA was fragmented using NEBNext dsDNA Fragmentase (New England Biolabs, Inc., Ipswich, MA, USA). Library preparation was performed using the Swift 2S Turbo DNA Library Kit (Swift Biosciences, Inc., USA) in accordance with the manufacturer’s instructions. Purification and size selection were performed using HiAccuBead (AccuGene, San Diego, CA, USA). Index polymerase chain reaction was performed using the Swift 2S Turbo Combinatorial Dual Indexing Primer Kit (Swift Biosciences, Inc.). Then, additional purification and size selection were conducted using HiAccuBead. Each sample’s library concentration was quantified using the Qubit dsDNA HS Assay Kit (Thermo Fisher Scientific, USA). The equimolar concentrations of each library (4 nM) were pooled and sequenced on the Illumina NovaSeq 6000 System (250-bp paired end).

The raw sequence data obtained from the NovaSeq system were processed in accordance with the methods of previous studies (31, 32). The adapter sequences were trimmed, and quality filtering was performed using Trimmomatic software (33). The paired-end sequences were merged using PEAR v.0.9.11 (34). The human-derived sequences in the metagenome data were identified and removed using BBMap with a reference human genome. Taxonomic profiling was conducted using Kraken2 (35), whereas functional annotation was performed using DIAMOND (36) against the Uniref90 database (37). Subsequently, the Uniref90 IDs were mapped to Kyoto Encyclopedia of Genes and Genomes (KEGG) Orthology (KO) terms. The reads per kilobase were normalized using the cumulative sum scaling method.

### Microbiome analysis

The potential contaminants during sampling and the experimental processes were identified and removed based on the sequence data from the negative control samples using the R package “decontam” (38). Decontamination was performed using the prevalence-based method in the “*isContaminant*” function with a strict threshold of 0.5. Species with >30% prevalence and a mean relative abundance of ≥0.001% were retained for further analysis to reduce false positives (39, 40). The alpha diversity, including the Shannon diversity index, was calculated using the “*alpha*” function in the R package “microbiome” (41). The differences in the microbiota among the samples were visualized using a nonmetric multidimensional scaling (NMDS) plot based on the Bary–Curtis dissimilarity.

The influence of covariates, including age, gender, treatment, treatment duration, clinical scores, and survey data, on microbiota was assessed using the “*envfit*” function in the R package “vegan” v.2.5-7. Each covariate’s effect size and statistical significance were determined and compared.

Multivariate Association with Linear Models (MaAsLin2) (42) was used to identify bacterial taxa that significantly differed between groups. The model coefficients, *P*-values, and adjusted *P*-values (*q*-values) were used for statistical interpretation. The inter- and intraindividual variations in the microbiome were compared using a box plot based on the Bray–Curtis dissimilarity.

A generalized linear model (GLM) was used to identify the association between taxonomic and functional features and clinical scores. The clinical scores were treated as the dependent variable, whereas the taxonomic and functional features were considered as the independent variables. Statistical significance was considered at *P* < 0.05.

The interspecies correlations were estimated using the Spearman correlation. The resulting correlation network was visualized using the R package “qgraph” (43). The node sizes were scaled according to PageRank centrality, which was calculated using the R package “igraph.” The edge thickness represented the Spearman correlation (rho), and only significant correlations (*P* < 0.05) were included in the network. The species with the highest PageRank values were designated as the keystone species in the network.

The features that were identified as significant in the GLM and Spearman correlation analyses were included in the mediation analysis. For the forward-effect hypothesis, the first model was fitted with taxonomic abundance and functional features as the treatment variable and mediator, respectively. The second model used the clinical score as the outcome variable to evaluate the association between the mediator and the treatment variable. These two models were used in the mediation analysis to estimate the average causal mediated effect (ACME) with the “*mediate*” function in the R package “mediation.” For the reverse-effect hypothesis, the first model was fitted with the clinical score and microbial abundance as the treatment variable and mediator, respectively. Meanwhile, the second model used the functional features as the outcome to assess their association with the mediator. Only the mediation results with ACME *P* < 0.05 for the forward effect and ACME *P* > 0.05 for the reverse effect were considered.

A Circos plot was used to visualize the relationships between key species and pathways in the third-level KO categories using the R packages “ggplot2,” “ggalluvial,” and “circlize.” These diagrams also illustrated the results of the mediation analysis, highlighting the microbial taxa that influenced the KO pathways and subsequently impacted the clinical score remission.

Procrustes analysis was performed to assess the congruence between the taxonomic and functional features of the microbiome using the “*protest*” function in the R package “vegan” with 999 permutations. The microbiota composition dissimilarity was calculated using the Bray–Curtis distance, whereas the functional feature distances were computed using the Hellinger method. An NMDS plot was generated to visualize the relationships between taxonomic and functional changes within each sample.

### *In vitro* co-culture assay to assess growth inhibition of *Pseudomonas putida*

To validate whether co-cultivation with *Streptococcus lividans* (ATCC 19,844) or *Edwardsiella tarda* (KCTC 12,267) inhibits the growth of *Pseudomonas putida* (KCTC 1751), we performed aerobic co-cultivation experiments. Three culture conditions were prepared in triplicate: (i) *P. putida* monoculture, (ii) *P. putida + S. lividans* co-culture, and (iii) *P. putida + E. tarda* co-culture. All strains were grown in Tryptic Soy Broth (TSB; BD, East Rutherford, NJ, USA).

For each condition, cultures were prepared in 20 mL TSB. Monocultures were inoculated at 1% (vol/vol). For co-cultures, each strain was inoculated at 1% (vol/vol) (i.e., 1% + 1% total inoculum). Cultures were incubated aerobically at 27°C with shaking at 200 rpm for 48 h. At 0, 12, 24, and 48 h after inoculation, 1 mL of broth was collected from each culture. Samples were centrifuged at 10,000 rpm for 10 min, and cell pellets were used for genomic DNA extraction using the DNeasy PowerSoil Pro Kit (Qiagen) according to the manufacturer’s instructions.

Growth of *P. putida* was quantified using species-specific quantitative real-time PCR (qPCR) with validated primers (in-house design): forward 5′-TTTGATGGCACGGTTGGCTA-3′ and reverse 5′-CGCCCATGATGCTCATGTTTT-3′. qPCR was performed on a Thermal Cycler Dice Real-Time System III (Takara Bio, Shiga, Japan) in 25 µL reactions containing 12.5 µL of 2 × TB Green Premix Ex Taq (Tli RNaseH Plus; Takara Bio), 2 µM of each primer, and 2 µL of 10-fold serially diluted DNA template or distilled water as a no-template control. Cycling conditions were 95°C for 30 s, followed by 40 cycles of 95°C for 5 s and 60°C for 30 s.

*P. putida* gene copy numbers were calculated by interpolating Ct values onto a standard curve generated from 10-fold serial dilutions (1 × 10^1^ to 1 × 10^7^ copies) of *P. putida* genomic DNA. The standard curve was constructed using DNA extracted from *P. putida* monocultures with colony-forming units (CFUs) determined by plating on TSB agar, and it showed high linearity (*R*^2^ ≥ 0.99).

### Statistical analysis

Pairwise comparisons between two groups were performed using the Wilcoxon rank-sum test. Dunn’s multiple comparison test was used to assess significant differences between two or more groups, and *P*-values were adjusted using the Benjamini–Hochberg procedure in the R package “dunn.test.” The beta diversity differences were visualized using NMDS plots based on the Bray–Curtis dissimilarity, and statistical significance was assessed using permutational multivariate analysis of variance (Adonis2 function in the R package “vegan”) with 999 permutations. The correlations between taxonomic and functional features and clinical scores, survey data, and treatment time points were evaluated using Spearman correlation analysis, which was performed using the “*rcorr.adjust*” function in the R package “Hmisc.” Results with *P* < 0.05 were considered statistically significant.

## RESULTS

### Dry eye symptoms improved in the CsA and control groups

The OS samples, clinical data, and survey responses were collected at three time points: before initiating eyedrop treatment (V1), after 4 weeks of treatment (V2), and after 12 weeks of treatment (V3) (Fig. S1). The clinical characteristics of the participants at each visit are summarized in Table 1. No significant differences in gender or age were observed between the treatment groups or across time points. Moreover, the clinical scores, symptom frequency, symptom severity, and comorbidities did not significantly differ between the treatment groups at any time point. However, the meibomian gland dysfunction (MGD) and TBUT significantly improved in the CsA and control groups following eyedrop treatment (*P* < 0.01). Furthermore, the OSDI showed significant improvement in the CsA group (*P* < 0.05).

**TABLE 1:** Clinical characteristics of the study participants^*a*^

Characteristic	Treatment						Significance (*P-*value)				
	NewHyalUni alone			CsA + NewHyalUni			Longitudinal changes^*b*^		Inter-treatment group^*c*^		
	V1	V2	V3	V1	V2	V3	NewHyalUni alone	CsA + NewHyalUni	V1	V2	V3
Gender (*n*, male/female)	1/12	1/9	1/7	3/10	3/6	3/5	1.000	0.881	0.593	0.311	0.569
Age (years old)	54.92 ± 2.54	54.90 ± 3.27	51.38 ± 2.15	55.38 ± 3.75	58.22 ± 6.67	56.75 ± 7.37	0.730	0.702	0.521	0.888	1.000
Clinical score											
OSDI	49.14 ± 5.96	34.51 ± 4.60	36.21 ± 7.17	53.54 ± 5.52	41.90 ± 5.12	30.35 ± 4.62	0.263	< 0.05	0.608	0.254	0.613
VAS (0–10)	3.00 ± 0.85	2.30 ± 0.58	2.88 ± 0.75	3.58 ± 0.76	3.33 ± 0.88	1.86 ± 0.43	0.693	0.312	0.545	0.186	0.263
MGD (right eye)	2.08 ± 0.18	1.30 ± 0.21	0.88 ± 0.23	2.31 ± 0.13	1.33 ± 0.17	1.00 ± 0.29	<0.01	<0.001	0.358	0.716	1.000
MGD (left eye)	2.08 ± 0.18	1.30 ± 0.21	0.75 ± 0.24	2.31 ± 0.13	1.33 ± 0.17	1.00 ± 0.29	<0.002	<0.001	0.358	0.716	0.574
TBUT (s, right eye)	3.92 ± 0.26	6.60 ± 0.52	7.25 ± 1.37	3.62 ± 0.27	8.11 ± 0.59	7.43 ± 1.15	<0.003	<0.001	0.302	0.113	0.850
TBUT (s, left eye)	4.00 ± 0.28	6.82 ± 0.55	7.25 ± 1.21	3.62 ± 0.27	8.11 ± 0.59	7.71 ± 1.01	<0.002	<0.001	0.236	0.967	0.753
F stain (right eye)	0.15 ± 0.15	0.45 ± 0.22	0.75 ± 0.30	0.33 ± 0.18	0.33 ± 0.24	0.57 ± 0.28	0.220	0.548	0.306	0.186	0.729
F stain (left eye)	0.15 ± 0.15	0.30 ± 0.13	0.50 ± 0.25	0.33 ± 0.14	0.33 ± 0.24	0.71 ± 0.27	0.339	0.436	0.172	0.219	1.000
Symptom frequency (per day)											
Dry (0–3)	2.15 ± 0.27	1.50 ± 0.27	2.00 ± 0.41	2.08 ± 0.21	1.67 ± 0.33	1.43 ± 0.19	0.357	0.229	0.662	0.713	0.178
Foreign body sensation (0–3)	1.46 ± 0.22	1.70 ± 0.26	1.75 ± 0.29	1.31 ± 0.26	0.78 ± 0.28	0.86 ± 0.32	0.709	0.477	0.723	0.086	0.076
Eye sensitivity (0–3)	1.31 ± 0.33	0.70 ± 0.30	1.38 ± 0.28	1.54 ± 0.31	1.22 ± 0.32	0.86 ± 0.24	0.369	0.362	0.615	0.283	0.214
Fatigue (0–3)	2.08 ± 0.24	1.30 ± 0.26	1.88 ± 0.45	2.15 ± 0.19	1.67 ± 0.37	1.57 ± 0.28	0.087	0.361	0.869	0.158	0.376
Ache (0–3)	0.85 ± 0.25	1.30 ± 0.33	0.88 ± 0.22	1.15 ± 0.30	1.00 ± 0.24	0.57 ± 0.28	0.758	0.344	0.500	0.970	0.376
Glare (0–3)	1.23 ± 0.32	1.20 ± 0.33	0.63 ± 0.25	1.38 ± 0.33	1.33 ± 0.37	0.86 ± 0.32	0.400	0.503	0.770	0.582	0.662
Symptom degree											
Dry (0–3)	2.08 ± 0.24	1.30 ± 0.21	1.88 ± 0.46	2.23 ± 0.28	1.89 ± 0.31	1.57 ± 0.28	0.293	0.316	0.725	0.136	0.663
Foreign body sensation (0–3)	1.54 ± 0.27	1.90 ± 0.31	2.00 ± 0.41	1.54 ± 0.35	1.33 ± 0.41	0.86 ± 0.32	0.610	0.458	0.979	0.422	0.060
Eye sensitivity (0–3)	1.54 ± 0.33	1.30 ± 0.42	1.38 ± 0.41	1.38 ± 0.31	1.22 ± 0.36	0.71 ± 0.27	0.950	0.374	0.751	1.000	0.299
Fatigue (0–3)	2.23 ± 0.23	1.20 ± 0.25	1.88 ± 0.42	1.92 ± 0.33	1.89 ± 0.31	1.29 ± 0.17	0.105	0.208	0.603	0.119	0.240
Ache (0–3)	1.00 ± 0.28	1.50 ± 0.40	1.25 ± 0.36	1.15 ± 0.34	1.33 ± 0.41	0.43 ± 0.28	0.813	0.184	0.890	0.884	0.108
Glare (0–3)	1.38 ± 0.35	0.90 ± 0.23	0.75 ± 0.24	1.54 ± 0.37	1.33 ± 0.50	0.86 ± 0.32	0.473	0.543	0.770	0.559	0.901
Comorbidities											
Liver disease (Y/N)	0/13 (0%)	0/11 (0%)	0/8 (0%)	2/11 (15.4%)	1/9 (10%)	1/7 (12.5%)	1.000	1.000	0.480	0.476	1.000
Kidney disease (Y/N)	0/13 (0%)	1/10 (9.1%)	0/8 (0%)	0/13 (0%)	0/10 (0%)	0/8 (0%)	0.606	1.000	1.000	1.000	1.000
Heart disease (Y/N)	1/12 (7.7%)	1/10 (9.1%)	0/8 (0%)	0/13 (0%)	1/9 (10%)	0/8 (0%)	1.000	0.594	1.000	1.000	1.000
Lung disease (Y/N)	0/13 (0%)	0/11 (0%)	0/8 (0%)	0/13 (0%)	1/9 (10%)	0/8 (0%)	1.000	0.594	1.000	0.476	1.000
Gastrointestinal disorder (Y/N)	2/11 (15.4%)	0/11 (0%)	0/8 (0%)	2/11 (15.4%)	1/9 (10%)	1/7 (12.5%)	0.492	1.000	1.000	0.476	1.000
Heartburn (Y/N)	4/9 (30.8%)	1/10 (9.1%)	1/7 (12.5%)	4/9 (30.8%)	1/9 (10%)	1/7 (12.5%)	0.533	0.624	1.000	1.000	1.000
Reflux esophagitis (Y/N)	4/9 (30.8%)	2/9 (18.2%)	2/6 (25%)	1/12 (7.7%)	0/10 (0%)	0/8 (0%)	0.886	1.000	0.322	0.476	0.467
Sleep disorder (Y/N)	3/10 (23.1%)	1/10 (9.1%)	0/8 (0%)	3/10 (23.1%)	2/8 (20%)	1/7 (12.5%)	0.527	1.000	1.000	0.587	1.000
Insomnia (Y/N)	2/11 (15.4%)	1/10 (9.1%)	0/8 (0%)	1/12 (7.7%)	1/9 (10%)	1/7 (12.5%)	0.780	1.000	1.000	1.000	1.000
Prefer spicy foods (Y/N)	4/9 (30.8%)	3/8 (27.3%)	2/6 (25%)	5/8 (38.5%)	7/3 (70%)	3/5 (37.5%)	1.000	0.720	1.000	0.086	1.000
Avoid cold foods (Y/N)	6/7 (46.2%)	3/8 (27.3%)	3/5 (37.5%)	3/10 (23.1%)	2/8 (20%)	0/8 (0%)	0.909	0.582	0.411	1.000	0.200
Headache (Y/N)	4/9 (30.8%)	1/10 (9.1%)	2/6 (25%)	1/12 (7.7%)	0/10 (0%)	0/8 (0%)	0.582	1.000	0.322	1.000	0.467

^*a*^ V1, before using eye drops; V2, after 4 weeks of using eye drops; V3, after 12 weeks of using eye drops; OSDI, ocular surface disease index; VAS, visual analog scale; MGD, meibomian gland dysfunction; TBUT, tear break-up time; F stain, fluorescein eye stain.

^*b*^ The significance of changes in each factor within the same treatment group over different sampling time points.

^*c*^ The significance of differences in each factor between treatment groups at the same sampling time point.

### The OS microbiome did not differ between the CsA and control groups

The OS microbiome was analyzed in 26 patients with dry eye undergoing treatment with CsA (*n* = 13) and NewHyalUni alone (control; *n* = 13). Seventy-three WMS data sets were analyzed, including 61 OS samples and 12 negative controls, which were collected across three time points (Fig. S1). To minimize false-positive results, the taxonomic identifications obtained from Kraken2 were filtered, and potential contaminants were removed using the Decontam pipeline. Considering that OS samples have a low biomass, sequencing-based studies are particularly susceptible to contamination, which can introduce bias (25). The potential contaminants that were introduced during the experimental procedures were identified using Decontam, based on the sequences detected in the negative controls, including the sampling membrane, DNA extraction kits, and sequencing library kits (Fig. S2). After removing false positives and contaminants, 39 species were identified as noncontaminants in the OS samples (Table S1).

No significant differences in the alpha and beta diversity of the OS microbiome were observed between the treatment groups at any time point, nor within each treatment group over the treatment course (*P* > 0.05; Fig. 1a and b). *Klebsiella pneumoniae*, *Pasteurella multocida*, *Staphylococcus aureus*, and *S. haemolyticus* were identified as the dominant species across all groups (Fig. 1c). Although species composition varied between groups and across time points, these differences were not statistically significant (*P* > 0.05).

The OS microbiome did not show statistically significant differences between the treatment groups. This finding may be because of the influence of the covariates on the OS microbiome composition. To assess this, the influence of covariates on OS microbiome variation was analyzed using the EnvFit model across all samples. Gender was identified as a significant factor influencing OS microbiome variation, with the microbiota differing between female and male participants (R^2^ = 0.095, *P* < 0.05; Fig. 1d and e). The OS microbiome diversity was higher in males than in females, and 10 species, including *Corynebacterium sementosum* and *C. striatum*, differed significantly between the two groups (*q* < 0.05 in MaAsLin2 results; Fig. S3).

The differences in the OS microbiome composition between females and males may lead to distinct covariate effects on the OS microbiome in each gender (Fig. 1f). The VAS, insomnia, and ache frequency significantly influenced the OS microbiome in females, whereas treatment was the primary influencing factor in males (*P* < 0.05). Although the OS microbiome in males varied according to treatment, the number of male participants (*n* = 1 for the control group and *n* = 3 for the CsA group) was insufficient for robust statistical analysis and conclusions. Therefore, subsequent analyses focused on the OS microbiome in females (*n* = 28 for the control group and *n* = 21 for the CsA group; OS sample numbers across V1–V3).

The effects of VAS scores and ache frequency on the OS microbiome in females were further evaluated using Spearman correlation analysis between the microbiota and each factor (Fig. S4a and b). However, no significant correlations were observed between the VAS scores or ache frequency and the OS microbiota, either in the overall sample set or at each time point (*P* > 0.05). Moreover, Bray–Curtis dissimilarity analysis indicated no significant difference in the microbiota between patients with and without insomnia (Fig. S4c). These findings indicate that although gender is a significant factor influencing OS microbiota variation, the effects of other covariates identified by EnvFit remain unclear.

### OS microbiome was significantly associated with improvements in clinical scores during treatment

**Fig 2:**
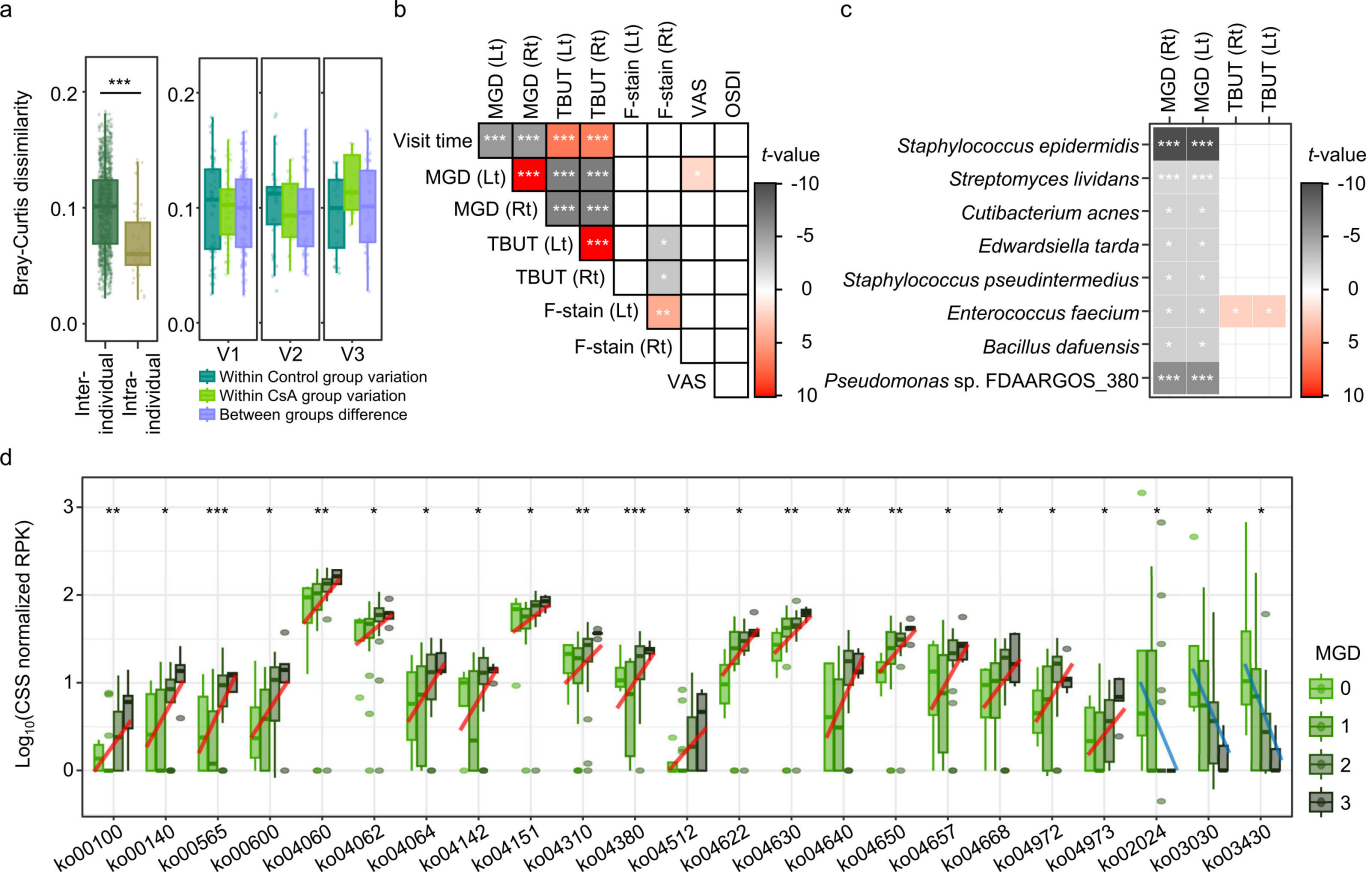
Individual variation in the OS microbiome and its associations with clinical scores. (**a**) The inter- and intraindividual variations in microbial composition were compared based on the Bray–Curtis dissimilarity. The differences between inter- and intraindividual variation were evaluated using the Wilcoxon rank-sum test. (**b**) The changes in the clinical scores over time were analyzed using a general linear model (GLM). (**c**) The GLM was also used to identify species that were significantly associated with the clinical scores. The *t*-values indicate the statistical significance of regression coefficients. (**d**) The microbiome functional features that were significantly associated with the MGD scores were identified based on Kyoto Encyclopedia of Genes and Genomes Orthology level 3. The significance was determined using Spearman correlation analysis. VAS, visual analog scale; MGD, meibomian gland dysfunction; TBUT, tear break-up time; OSDI, ocular surface disease index; F-stain, fluorescein eye stain. ***P < 0.001, **P < 0.01, *P < 0.05.

The individual variations in the OS microbiome may obscure the effects of host covariates and treatments. Therefore, the individual variation in the OS microbiota was analyzed. The interindividual variation of the OS microbiome was significantly higher than the intraindividual variation over time (*P* < 0.001; Fig. 2a). In addition, at each visit, the between-group difference was not significantly greater than the within-group inter-individual variation (*P* > 0.05). This finding suggests that the substantial variability in the OS microbiome between individuals may contribute to the unclear effects of host covariates and treatments.

Although the interindividual variation in the OS microbiota was higher than the intraindividual variation during the treatments, the dry eye symptoms improved in the CsA and control groups. A GLM analysis was performed to assess the association between time-dependent improvements in clinical scores and OS microbiota alterations during treatment. The MGD and TBUT significantly improved over the treatment course (*P* < 0.001; Fig. 2b), which were consistent with the results presented in Table 1. Moreover, the changes in the MGD scores were significantly correlated with the TBUT scores (*P* < 0.001).

The GLM method was also used to identify microbial species that were significantly associated with changes in the MGD and TBUT scores (Fig. 2c). The relative abundances of eight species (*Staphylococcus epidermidis*, *Streptomyces lividans*, *Cutibacterium acnes*, *Edwardsiella tarda*, *Staphylococcus pseudintermedius*, *Enterococcus faecium*, *Bacillus dafuensis*, and *Pseudomonas* sp. FDAARGOS_380) were significantly correlated with the MGD scores. Among them, *E. faecium* was also significantly correlated with the TBUT scores (*P* < 0.05). These findings suggest that the OS microbiota is more strongly associated with the MGD than with TBUT scores.

Alterations in the OS microbiota associated with improvements in the MGD and TBUT scores may alter the functional role of the OS microbiome during treatment. To investigate this, the metabolic pathways that were significantly associated with the MGD and TBUT scores were identified based on the functional genes in the OS microbiome using Spearman correlation and GLM analyses (Fig. S5). The normalized abundance of microbiome genes in the 64 pathways was significantly correlated with the MGD and TBUT scores during treatment in at least one of the statistical analyses (*P* < 0.05). Among them, the changes in the normalized abundance of microbiome genes in 23 pathways associated with the MGD scores were consistently identified in both statistical analyses (*P* < 0.05; Fig. 2d).

Of these pathways, the microbiome genes in 20 pathways increased with the MGD score, whereas those in three pathways decreased. Notably, the microbiome genes involved in inflammatory cytokine-mediated signaling and cell-death-related pathways were significantly associated with MGD scores (*P* < 0.05). These pathways included the PI3K-Akt signaling pathway (ko04151), NF-kappa B signaling pathway (ko04064), chemokine–cytokine receptor interaction (ko04062), Wnt signaling pathway (ko04310), Jak-STAT signaling pathway (ko04630), natural killer-cell-mediated cytotoxicity (ko04650), IL-17 signaling pathway (ko04657), and tumor necrosis factor (TNF) signaling pathway (ko04668). These findings suggest that the OS microbiome may contribute to the improvement of inflammation-related MGD scores.

### Microbe–microbe interactions within the OS microbiome changed during the treatment of dry eye symptoms

**Fig 3:**
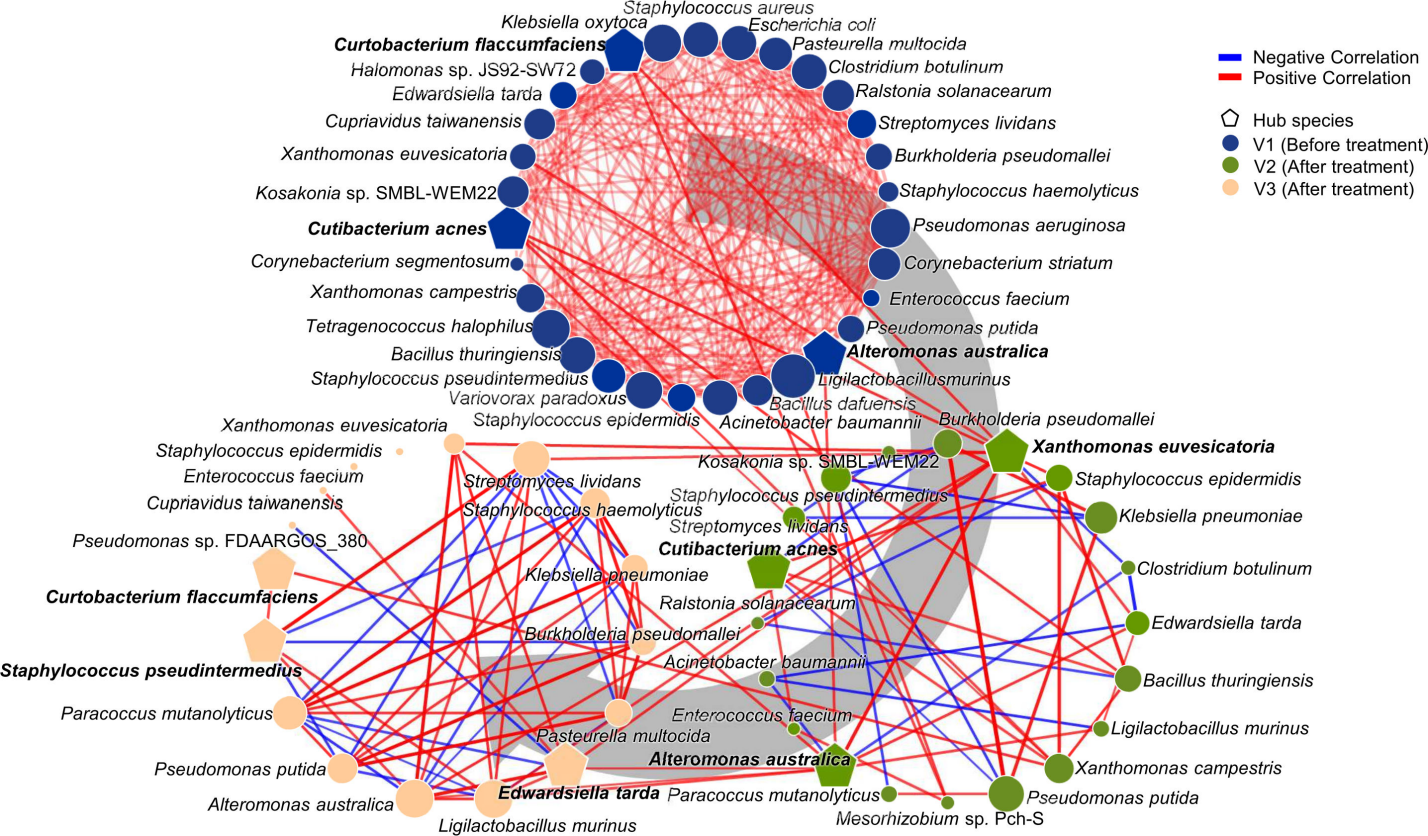
Network analysis of interspecies interactions in the OS microbiota over the treatment course. Microbial interaction networks were constructed to identify changes in interspecies correlations over time. The V1 group (pretreatment, dark blue) is displayed at the top, the V2 group (after 4 weeks of treatment, green) is displayed on the right, and the V3 group (after 12 weeks of treatment, light orange) is displayed on the left. Positive correlations are indicated by red edges, whereas negative correlations are indicated by blue edges. The edge thickness reflects the Spearman correlation coefficient (|ρ| > 0.7, *P* < 0.05). Each node represents a species, with the node size scaled according to the PageRank values. The top three hub species in each network are marked with pentagon symbols.

The species that were significantly associated with the MGD score changed during the treatments. These changes might have been influenced by microbe–microbe interactions within the OS microbiome. Therefore, microbial interactions in the OS microbiome were analyzed using network analysis (Fig. 3). The keystone species, defined as hub nodes in the network, were identified using the PageRank algorithm (denoted by the pentagon symbol).

Microbial interactions were more complex and predominantly positive before treatment (V1) compared with those observed after treatments (V2 and V3). *Curtobacterium flaccumfaciens*, *C. acnes*, and *Alteromonas australica* were identified as keystone species at V1. The predominance of positive correlations among microbes, including keystone species, suggests that the diverse microbes, including inflammation-associated species in the OS microbiome, can coexist and persist prior to treatment.

Following treatment, microbial interactions became less complex, with an increase in negative correlations among microbes, including keystone species. *Xanthomonas euvesicatoria*, *A. australica*, and *C. acnes* were identified as the keystone species at V2, whereas *E. tarda*, *S. pseudintermedius*, and *C. flaccumfaciens* emerged as the keystone species at V3. Notably, *C. acne* and *A. australica* were consistently identified as the keystone species at V1 and V2, whereas *C. flaccumfaciens* was a keystone species at V1 and V3. These results indicate that microbial interactions were regulated by persistent keystone species from V1 to V2, whereas distinct microbial interactions driven by different keystone species were associated with improvements in dry eye symptoms. These findings also suggest that microbial interactions within the OS microbiome shifted during treatment, which coincided with improvements in the MGD score. Notably, the reduction in the complex positive correlations and the increase in the negative correlations among microbes may have contributed to the suppression of inflammation-associated microbes, aligning with improvements in dry eye symptoms.

### The OS microbiome could mediate the improvement of MGD scores through its potential functions

The OS microbiome underwent changes in microbial interactions during treatment. Moreover, these interactions shifted in accordance with improvements in the MGD score. Therefore, alternative analytical approaches should be used to assess the improvement of dry eye symptoms through microbial changes in the OS microbiome. A mediation analysis incorporating altered microbial taxa, potential microbial functions inferred from the whole metagenome, and MGD scores was performed to determine whether improvements in clinical scores were mediated by alterations in the OS microbiome.

**Fig 4:**
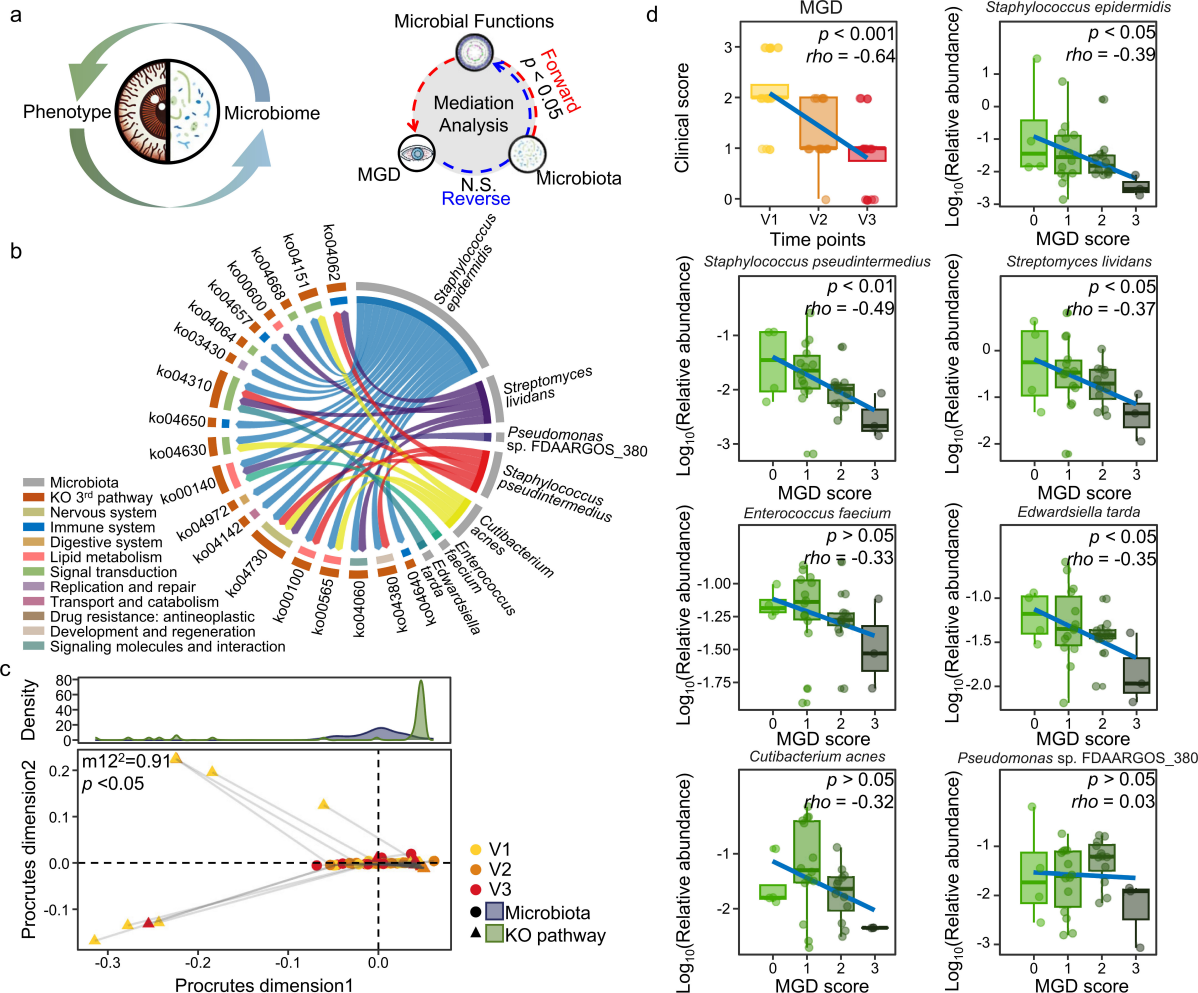
Mediation analysis to investigate potential causal relationships among the taxonomic and functional features of the OS microbiome and MGD scores. (**a**) Schematic overview of the hypothesized causal relationships between the OS microbiome and MGD scores. Mediation analysis was performed to identify significant mediating and causal effects among microbial taxa, microbial functions, and MGD scores. Only results with a significant ACME in the forward direction (*P* < 0.05) and a non-significant reverse ACME (*P* > 0.05) were considered. (**b**) Circos plot displaying significant mediation pathways, highlighting associations among microbial taxa, functional pathways, and MGD scores. (**c**) Procrustes analysis assessing whether shifts in microbiota composition are reflected in corresponding changes in the microbial functional profiles. The analysis is based on the correlation derived from the symmetric sum of squares (m12^2^). (**d**) Spearman correlation analysis between MGD scores and key species that were identified through mediation analysis.

The species and microbiome genes significantly associated with MGD scores, which were identified through Spearman correlation and GLM analysis (Fig. 2c; Fig. S5), were used for the mediation analysis. The ACME was assessed to evaluate the relationship’s directionality. A significant ACME (*P* < 0.05) for the forward effect (red arrow in Fig. 4a), where altered microbiota influenced the MGD score through their functions, and a non-significant ACME (*P* > 0.05) for the reverse effect (blue arrow in Fig. 4a), where the MGD score influenced microbiome functional changes via altered microbiota, were considered indicative of a potential causal relationship between OS microbiome changes and clinical improvement.

Seven species were identified as mediating effects on 21 pathways with significant ACME (*P* < 0.05; Fig. 4b). Among them, *S. epidermidis* was associated with 19 pathways, suggesting that it may be the most influential species contributing to MGD score improvement through microbiome function. *S. epidermidis* was linked to pathways involved in the immune system, lipid metabolism, signal transduction, and signaling molecules and interactions. *S. lividans* and *S. pseudintermedius* were also associated with pathways related to the immune system, lipid metabolism, and signal transduction. *C. acnes* was linked to lipid metabolism and signal transduction, whereas *E. faecium* was associated with lipid metabolism. *E. tarda* and *Pseudomonas* sp. FDAARGOS_380 were linked to signal transduction and lipid metabolism, respectively, although their associations with other pathways were less extensive compared with those of other species.

Procrustes analysis was used to further evaluate the potential functional changes in the OS microbiome mediated by the selected species from the mediation analysis (Fig. 4c). Procrustes analysis enables the statistical comparison of two or more matrices by analyzing shape similarities and aligning corresponding data points across data sets. The alterations in metabolic pathways based on KO were significantly correlated with changes in microbial composition (m12^2^ = 0.91, *P* < 0.05, 999 permutations).

Spearman correlation analysis was performed to further validate the associations between the selected species and the MGD score (Fig. 4d). The MGD scores decreased over the treatment course (*P* < 0.001), and four species (*S. epidermidis*, *S. pseudintermedius*, *S. lividans,* and *E. tarda*) were negatively correlated with the MGD score (*P* < 0.05). These findings, which were obtained through multiple analytical approaches, indicate that shifts in these four species within the OS microbiome during treatment contribute to the improvement of the MGD score through their functional roles.

### The suppression of inflammation-related species by mediating species might contribute to the improvement of the MGD scores during treatment

*S. epidermidis*, *S. pseudintermedius*, *S. lividans,* and *E. tarda* can contribute to the improvement of MGD scores through microbiome functions. Among them, *S. pseudintermedius* and *E. tarda* were identified as keystone species in microbial interactions at V3 (Fig. 3). Spearman correlation analysis was performed to assess the potential direct association between the MGD scores and keystone species at other time points (Fig. S6). Although the relative abundances of keystone species increased during treatment, only two species, *C. flaccumfaciens* and *A. australica*, showed statistically significant changes (*P* < 0.05). Furthermore, a significant association with MGD scores was observed only for *C. flaccumfaciens*, a keystone species at V3 (*P* < 0.01). These findings suggest that the keystone species may not be directly involved in the alleviation of dry eye symptoms, except for *C. flaccumfaciens*. Instead, they may function as hubs or regulators influencing the microbiome dynamics associated with improved MGD scores.

**Fig 5:**
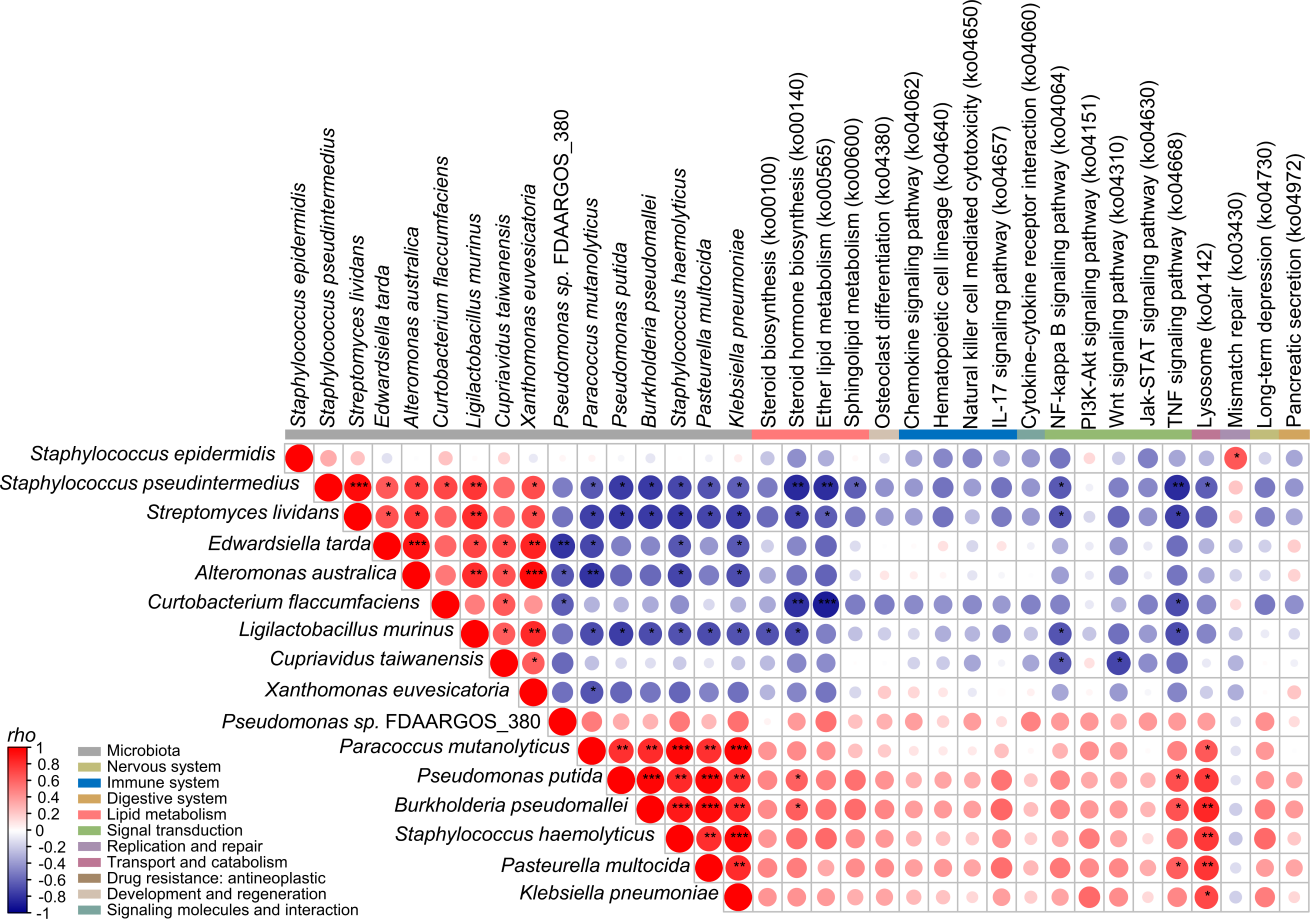
Correlation analysis among key species that were identified through mediation analysis, keystones from network analysis, and significant microbial functions associated with the MGD score. The Spearman correlation coefficients among these features are visualized in a correlogram. Positive correlations are indicated by red circles, whereas negative correlations are indicated by blue circles. The size of each circle corresponds to the strength of the correlation (Spearman′s ρ). ***P < 0.001, **P < 0.01, *P < 0.05.

To further investigate the role of key species that were identified in the mediation analysis and keystone species in the network analysis in improving MGD scores via microbiome functions, the considerably correlated species and KO pathways were analyzed (Fig. 5). The key species that were identified in the mediation analysis and the keystone species were positively correlated with each other and negatively correlated with six species: *Paracoccus mutanolyticus*, *Pseudomonas putida*, *Burkholderia pseudomallei*, *Staphylococcus haemolyticus*, *Pasteurella multocida*, and *Klebsiella pneumoniae*. These six species were positively correlated with inflammation- and cytokine-related pathways. These results indicate that the key species identified in the mediation analysis may contribute to the improvement of MGD scores by suppressing inflammation-related species. To validate whether prioritized taxa could modulate inflammation-associated species, we performed co-culture assays and observed growth inhibition of *P. putida* by *S. lividans* and *E. tarda* (Fig. S7).

## DISCUSSION

The influence of the OS microbiome on dry eye disease during treatment with eye drops was analyzed after the removal of potential contaminants. Although the OS microbiome did not show significant differences based on the type of eye drop used, the improvements in dry eye symptoms were associated with shifts in the OS microbiome during treatment. The changes in microbiome functions, which were driven by alterations in the OS microbiota, may mediate the alleviation of MGD scores, as indicated by multiple analyses. Notably, these effects could be attributed to the changes in microbial interactions within the OS microbiome during treatment. Therefore, the OS microbiome may play a role in the pathophysiology of dry eye disease.

Previous studies have reported OS microbiome changes in response to the use of eye drops and treatment duration (44, 45). The OS microbiome was found to differ significantly between individuals who used eye drops and those who did not. In the present study, NewHyalUni and CsA, which are widely used eye drops for treating dry eye, were evaluated. NewHyalUni helps rehydrate the corneal moisture layer and improves dry eye symptoms (46, 47), whereas CsA alleviates dry eye by reducing corneal surface inflammation and preventing the inflammation-induced loss of goblet cells (48). The changes in the moisture levels and immune response in the eye may lead to alterations in the OS microbiome. However, no significant differences in the OS microbiome were observed between the NewHyalUni alone and CsA combination treatment groups or across treatment durations. This discrepancy between the present study and previous studies may be attributed to the analysis pipeline, which included the removal of potential contaminants from the whole metagenome data set, and the influence of host covariates on OS microbiome variations.

The influence of host factors, including age and sex, on the OS microbiome remains inconsistent across previous studies. While some studies have reported significant differences in the OS microbiome based on age but not sex (7, 24, 49), others have observed gender-based differences, which are likely influenced by hormonal variations (50, 51). In the present study, gender was a significant factor affecting OS microbiome variation, and the clinical scores influenced OS microbiome variation differently between males and females, as indicated by the EnvFit model. A plausible biological contributor to this gender-associated variation is endocrine regulation. Sex steroids can modulate microbiome and mucosal immune responses, and experimental studies have shown that hormonal perturbations can shift microbiota (52, 53). Moreover, the ocular surface is hormonally responsive, and endocrine factors have been implicated in tear film homeostasis, glandular function, and inflammatory regulation in dry eye disease (54). However, there were no significant correlations between host covariates (e.g., VAS, insomnia, and ache frequency) and OS microbiome variation. This may be because of the greater-than-expected individual variation in the OS microbiome.

Despite the influence of eye drop treatment on OS microbiome alterations, dry eye symptoms improved in both treatment groups. According to the results of the GLM analysis, clinical score improvements during treatment were significantly associated with microbial changes in the OS microbiome. Notably, the improvements in the MGD and TBUT scores were significant throughout the treatment period, with the former showing a stronger association with taxonomic and functional changes in the OS microbiome compared with the latter. MGD is recognized as the primary cause of evaporative dry eye disease, with higher prevalence rates reported in Asia than in other regions (55–57). MGD is characterized by chronic, diffuse abnormalities of the meibomian gland structures, terminal duct obstruction, and meibum secretion alterations (58). The key pathogenic factors of MGD include the proliferation of inflammation-associated microbes, meibomian gland blockage, and gland dropout (59). Therefore, the significant association between OS microbiome alterations and alleviated MGD scores during treatment may be attributed to the suppression of inflammation-related microbes in the OS microbiome. The results of the mediation analysis further suggested that MGD improvement may be driven by OS microbiome alterations through microbiome functions. Moreover, inflammation-related microbiome functions exhibited significant changes in accordance with the MGD scores.

OS microbiome alterations may result from changes in microbial interactions during treatment. Complex positive correlations among microbes were observed before treatment, which were reduced following treatment. These findings suggest that the inflammation-associated microbes proliferated in the OS of patients with dry eye disease through complex positive correlations. However, these microbes were suppressed during treatment, likely because of negative correlations among microbes. Notably, the keystone species exhibited gradual changes throughout treatment, and the regulators of microbial interactions differed at the V3 time point, when dry eye symptoms showed improvement. The results of mediation analysis revealed that the keystone species at V1 and V2 did not significantly influence the improvement of MGD scores, whereas the key species that were identified in the mediation analysis and the keystone species at V3 were associated with the improvement of MGD scores. These species were negatively correlated with inflammation-related pathways, likely because of the suppression of inflammation-associated species, including *P. putida*, *S. haemolyticus*, and *K. pneumoniae*. Although the steroid hormone biosynthesis, TNF signaling, and lysosome pathways were significantly correlated with these species in the present study, various other inflammation-related pathways, including the chemokine signaling pathway, cytokine–cytokine receptor interactions, and the NF-kappa B signaling pathway, were also positively correlated with them.

According to a previous study, *K. pneumoniae, Pseudomonas*, and *Staphylococcus* species are associated with various ocular infections (60). Therefore, the positive correlation between these species and the inflammation-related pathways in the present study may be linked to the MGD scores in dry eye disease. Steroid hormones play an important role in maintaining the morphology and function of the OS (54), and hormonal imbalance has been implicated in the onset of dry eye disease, particularly increasing the risk in females (61). Elevated TNF-alpha levels are known to contribute to dry eye disease in human and mouse models (62, 63). The binding of TNF to TNF-R1 triggers intracellular signaling events, which ultimately activate major transcription factors, including NF-kappa B (64). Moreover, lysosomes are present in ocular tissues, and their dysfunction may contribute to the development of ocular diseases, including dry eye disease (65). These findings suggest that microbiome alterations following dry eye treatment may reduce inflammation-related microbes, thereby mediating the improvement of MGD scores. These findings also support the hypothesis that inflammation caused by OS microbes plays a role in the pathogenesis of dry eye disease.

Several limitations should be noted. The sample size was modest, and the number of male participants was limited, restricting the generalizability of gender-associated variations. Circulating sex hormone levels and endocrine-related variables (e.g., menopausal status or hormone therapy) were not assessed, precluding direct evaluation of hormone–microbiome relationships. Although a 12-week follow-up is commonly sufficient to capture the expected clinical response to topical cyclosporine A, longer follow-up would be required to determine the durability of microbiome changes and whether trajectories diverge by treatment group or by the magnitude of symptom improvement; however, extending follow-up beyond the routine treatment is operationally challenging because post-treatment clinic re-attendance is difficult to ensure in real-world practice. Finally, given the low-biomass nature of OS samples, stringent decontamination and quality-control procedures were applied, but residual technical variability cannot be fully excluded. Larger, sex-balanced cohorts with longer follow-up and integrative multi-omics analyses will be needed to validate and extend these findings. In conclusion, the OS microbiome in patients with dry eye disease may contribute to disease pathogenesis through its association with key pathways, including the steroid hormone biosynthesis, TNF signaling, and lysosome pathways. The alterations in the OS microbiome and changes in microbial interactions were observed alongside alleviated MGD scores during CsA and NewHyalUni treatment. Key species, including *S. epidermidis*, *S. pseudintermedius*, *S. lividans,* and *E. tarda*, may contribute to the improvement of MGD scores by suppressing inflammation-associated species through microbiome function modulation. Although further studies are needed to validate these findings, our results suggest that the modulation of the OS microbiome may aid in the treatment or prevention of dry eye disease.

## Data Availability

A STORMS (Strengthening The Organizing and Reporting of Microbiome Studies) checklist (66) is available at https://doi.org/10.5281/zenodo.15421760. The sequencing data obtained from this study are available in the EMBL SRA database under the study number PRJEB82823.

## SUPPLEMENTAL MATERIAL

Supplemental material msystems.00104-26-s0001.pdf Figs. S1 to S7; Table S1.

ASM does not own the copyrights to Supplemental Material that may be linked to, or accessed through, an article. The authors have granted ASM a non-exclusive, world-wide license to publish the Supplemental Material files. Please contact the corresponding author directly for reuse.
