# Supplementary material for: Potential role of the ocular surface microbiome in dry eye: microbial interactions and symptom alleviation
Source: mSystems. 2026 Mar 23;11(4):e00104-26. doi: 10.1128/msystems.00104-26 (PMC13098248; doi:10.1128/msystems.00104-26)
Supplement: Supplemental material — Figs. S1 to S7; Table S1. [file msystems.00104-26-s0001.pdf]

Supplementary Information

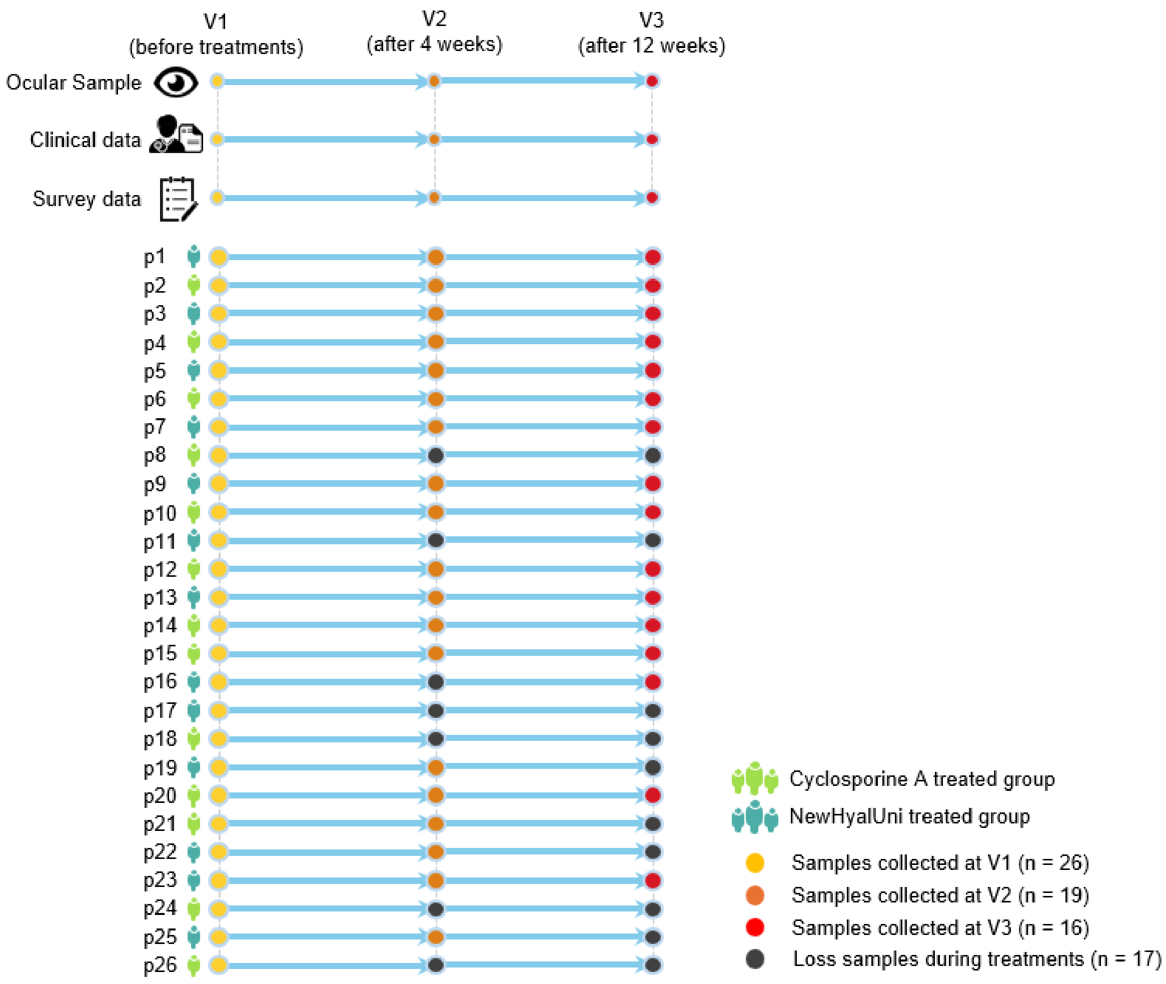

Fig. S1. Overview of the sampling strategy for the individual ocular surface (OS) samples included in this study.

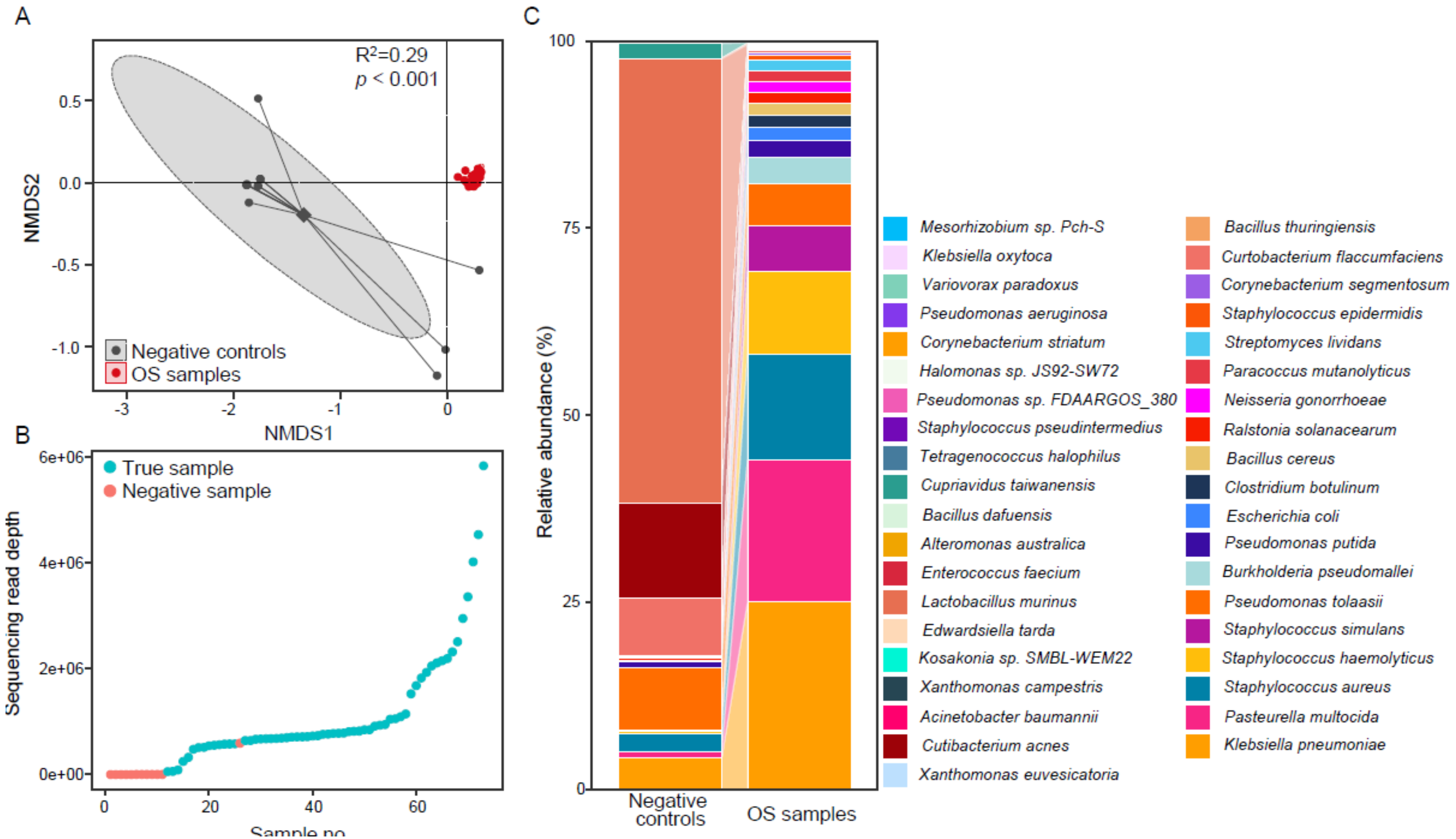

Fig. S2. Detection and removal of potential contaminant sequences using the Decontam pipeline. (A) Differences in the microbiota composition between ocular surface (OS) samples and negative controls. Statistical significance was assessed using permutational multivariate analysis of variance. (B) Sequencing read depth for each sample. (C) Comparison of the microbiota composition at the species level between the OS samples and the negative controls.

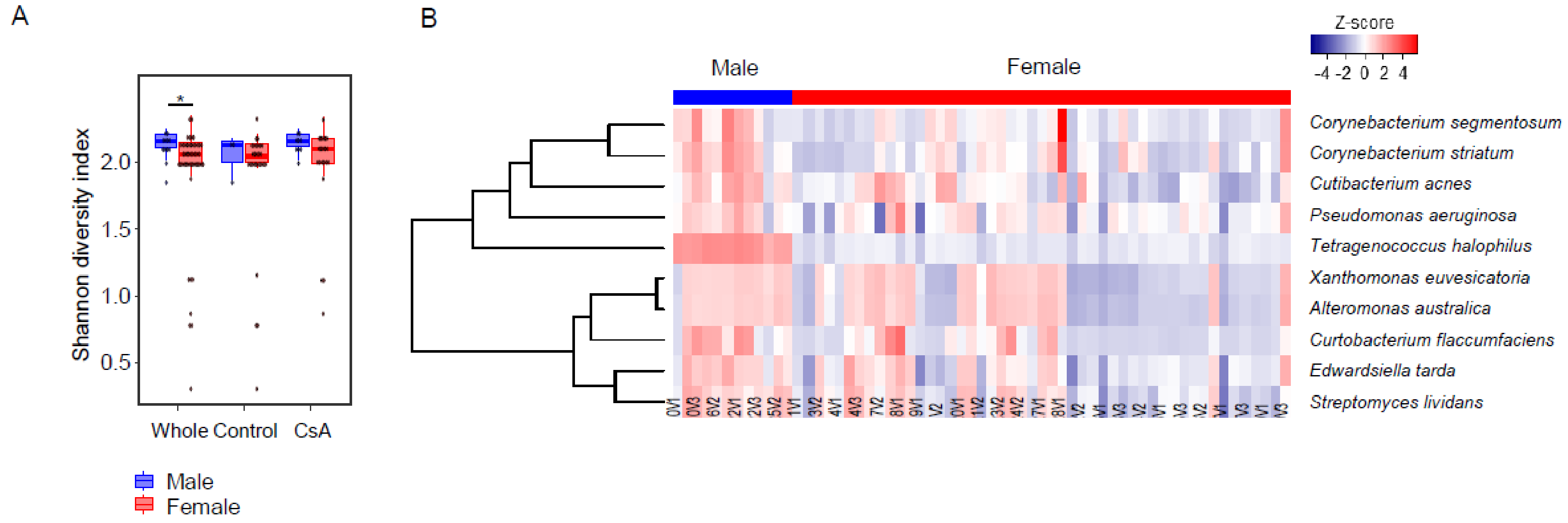

Fig. S3. Comparison of the ocular surface (OS) microbiota between males and females. (A) The microbial diversity between males and females was compared. The *p*-values were calculated using the Wilcoxon rank-sum test. (B) Heatmap analysis of significantly different species between males and females. ***$p < 0.001$, **$p < 0.01$, *$p < 0.05$.

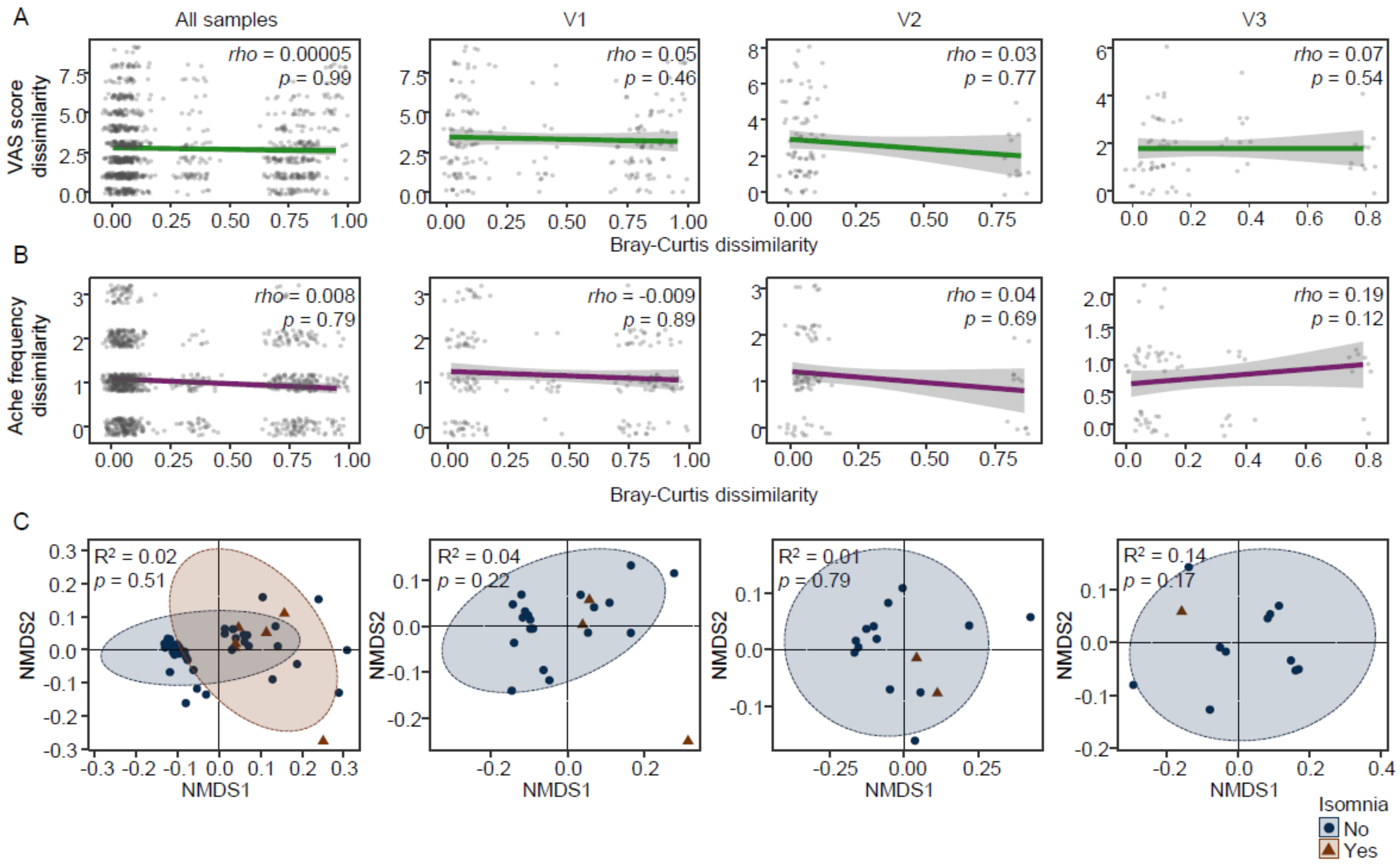

Fig. S4. Correlations between ocular surface microbiota and clinical characteristics at each time point. Spearman correlation analysis between microbiota dissimilarity (based on the Bray–Curtis distance) and clinical variables, including visual analogue scale (VAS) scores (A) and ache frequency (B). No significant correlations were observed. (C) Nonmetric multidimensional scaling (NMDS) plots comparing the microbiota composition according to the presence or absence of insomnia. Statistical significance was evaluated using permutational multivariate analysis of variance.

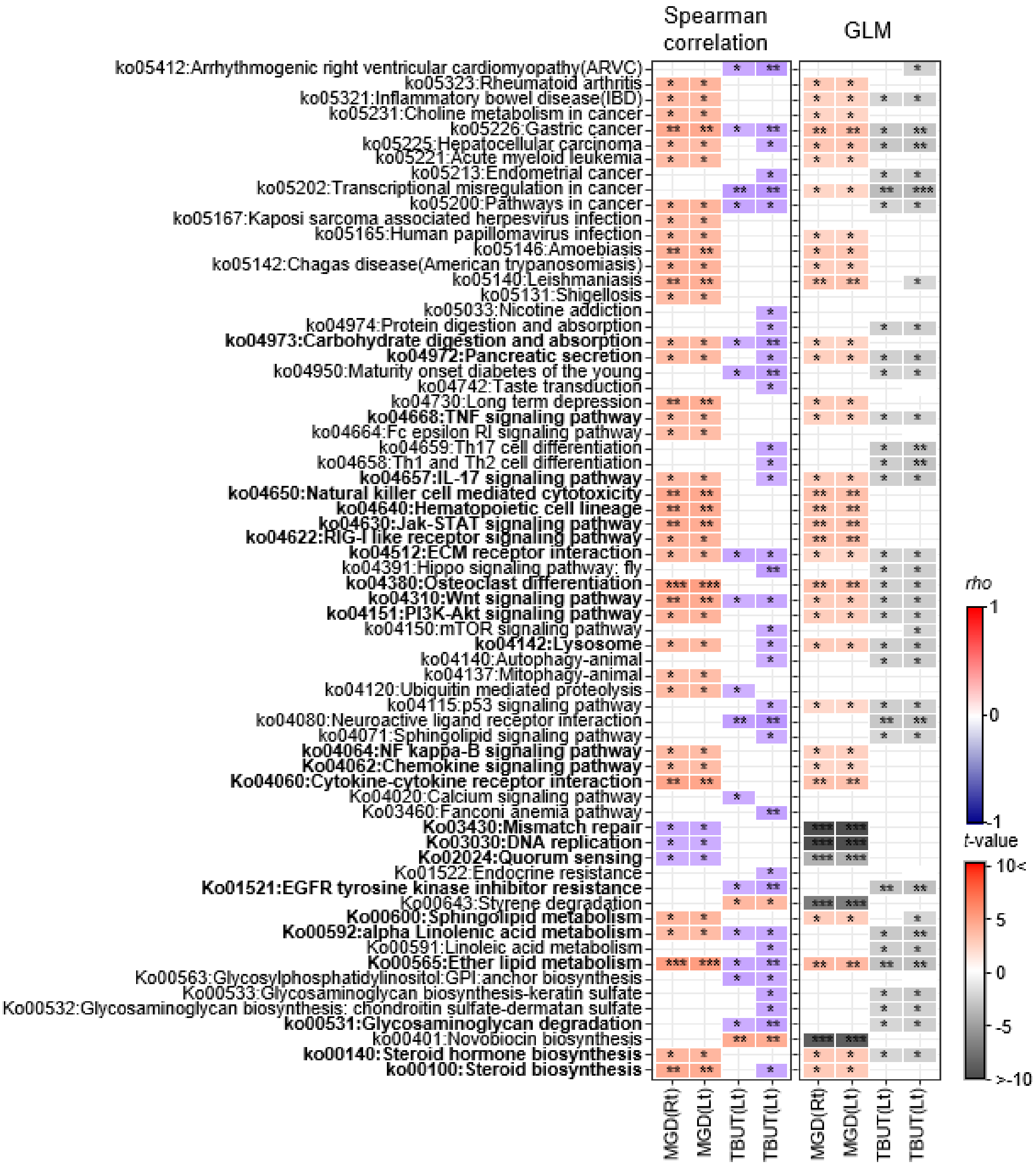

Fig. S5. Associations between clinical scores and functional features of the ocular surface microbiome. Spearman correlation analysis and generalized linear model (GLM) analysis were performed to evaluate the associations between clinical parameters and microbial functional profiles based on Kyoto Encyclopedia of Genes and Genomes Orthology (KO). MGD, meibomian gland dysfunction; TBUT, tear break-up time. ***$p < 0.001$, **$p < 0.01$, *$p < 0.05$.

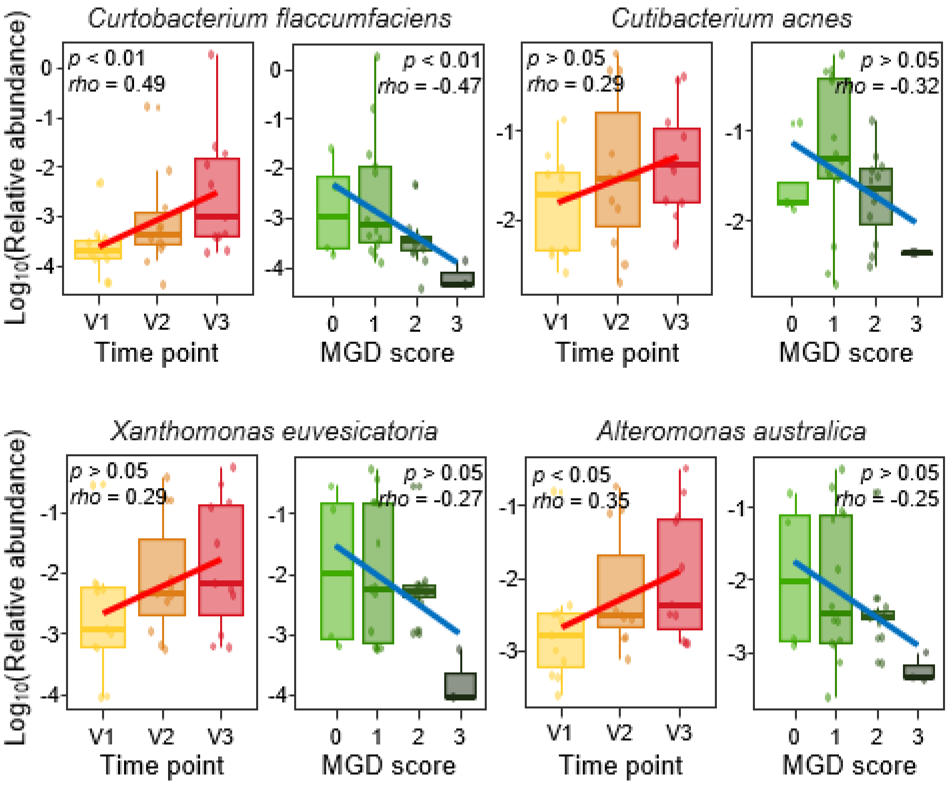

Fig S6. Associations between keystone species, treatment duration, and meibomian gland dysfunction (MGD) scores. Statistical significance was evaluated using Spearman's correlation analysis.

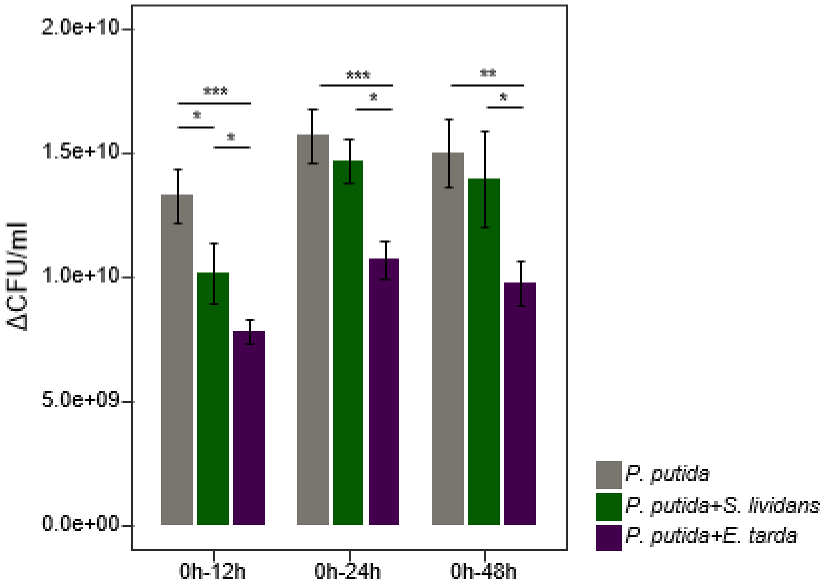

Fig S7. Co-cultivation qPCR validation of growth inhibition. *Pseudomonas putida* (KCTC 1751) was cultured alone or co-cultured with *Edwardsiella tarda* (KCTC 12267) or *Streptomyces lividans* (ATCC 19844) aerobically in TSB at 27°C. Samples were collected at 0, 12, 24, and 48 h. *P. putida* abundance was quantified by species-specific quantitative real-time PCR (qPCR) using P. putida-specific primers. Bars indicate changes from baseline (ΔCFU/mL) at 12, 24, and 48 h realative to 0 h (mean ± standard deviation). ***$p < 0.001$, **$p < 0.01$, *$p < 0.05$.

Table S1. List of noncontaminant species that were identified in ocular surface sequencing data after removal of false positives and contaminants.

Phylum	Class	Order	Family	Genus	Species	Mean relative abundance (%)	Decontam results
Proteobacteria	Alphaproteobacteria	Rhizobiales	Phyllobacteriaceae	*Mesorhizobium*	*Mesorhizobium* sp. Pch-S	0.001	Non-contaminant
Proteobacteria	Gammaproteobacteria	Enterobacterales	Enterobacteriaceae	*Klebsiella*	*Klebsiella oxytoca*	0.002	Non-contaminant
Proteobacteria	Betaproteobacteria	Burkholderiales	Comamonadaceae	*Variovorax*	*Variovorax paradoxus*	0.004	Non-contaminant
Proteobacteria	Gammaproteobacteria	Pseudomonadales	Pseudomonadaceae	*Pseudomonas*	*Pseudomonas aeruginosa*	0.012	Non-contaminant
Actinobacteria	Actinobacteria	Corynebacteriales	Corynebacteriaceae	*Corynebacterium*	*Corynebacterium striatum*	0.021	Non-contaminant
Proteobacteria	Gammaproteobacteria	Oceanospirillales	Halomonadaceae	*Halomonas*	*Halomonas* sp. JS92-SW72	0.047	Non-contaminant
Proteobacteria	Gammaproteobacteria	Pseudomonadales	Pseudomonadaceae	*Pseudomonas*	*Pseudomonas* sp. FDAARGOS_380	0.05	Non-contaminant
Firmicutes	Bacilli	Bacillales	Staphylococcaceae	*Staphylococcus*	*Staphylococcus pseudintermedius*	0.051	Non-contaminant
Firmicutes	Bacilli	Lactobacillales	Enterococcaceae	*Tetragenococcus*	*Tetragenococcus halophilus*	0.054	Non-contaminant
Proteobacteria	Betaproteobacteria	Burkholderiales	Burkholderiaceae	*Cupriavidus*	*Cupriavidus taiwanensis*	0.055	Non-contaminant
Firmicutes	Bacilli	Bacillales	Bacillaceae	*Bacillus*	*Bacillus dafuensis*	0.057	Non-contaminant
Proteobacteria	Gammaproteobacteria	Alteromonadales	Alteromonadaceae	*Alteromonas*	*Alteromonas australica*	0.066	Non-contaminant
Firmicutes	Bacilli	Lactobacillales	Enterococcaceae	*Enterococcus*	*Enterococcus faecium*	0.068	Non-contaminant
Firmicutes	Bacilli	Lactobacillales	Lactobacillaceae	*Ligilactobacillus*	*Lactobacillus murinus*	0.07	Non-contaminant
Proteobacteria	Gammaproteobacteria	Enterobacterales	Hafniaceae	*Edwardsiella*	*Edwardsiella tarda*	0.078	Non-contaminant
Proteobacteria	Gammaproteobacteria	Enterobacterales	Enterobacteriaceae	*Kosakonia*	*Kosakonia* sp. SMBL-WEM22	0.092	Non-contaminant
Proteobacteria	Gammaproteobacteria	Xanthomonadales	Xanthomonadaceae	*Xanthomonas*	*Xanthomonas campestris*	0.095	Non-contaminant
Proteobacteria	Gammaproteobacteria	Pseudomonadales	Moraxellaceae	*Acinetobacter*	*Acinetobacter baumannii*	0.106	Non-contaminant
Actinobacteria	Actinobacteria	Propionibacteriales	Propionibacteriaceae	*Cutibacterium*	*Cutibacterium acnes*	0.115	Non-contaminant
Proteobacteria	Gammaproteobacteria	Xanthomonadales	Xanthomonadaceae	*Xanthomonas*	*Xanthomonas euvesicatoria*	0.115	Non-contaminant
Firmicutes	Bacilli	Bacillales	Bacillaceae	*Bacillus*	*Bacillus thuringiensis*	0.142	Non-contaminant

Actinobacteria	Actinobacteria	Micrococcales	Microbacteriaceae	*Curtobacterium*	*Curtobacterium flaccumfaciens*	0.236	Non-contaminant
Actinobacteria	Actinobacteria	Corynebacteriales	Corynebacteriaceae	*Corynebacterium*	*Corynebacterium segmentosum*	0.415	Non-contaminant
Firmicutes	Bacilli	Bacillales	Staphylococcaceae	*Staphylococcus*	*Staphylococcus epidermidis*	0.653	Non-contaminant
Actinobacteria	Actinobacteria	Streptomycetales	Streptomycetaceae	*Streptomyces*	*Streptomyces lividans*	1.327	Non-contaminant
Proteobacteria	Alphaproteobacteria	Rhodobacterales	Rhodobacteraceae	*Paracoccus*	*Paracoccus mutanolyticus*	1.442	Non-contaminant
Proteobacteria	Betaproteobacteria	Neisseriales	Neisseriaceae	*Neisseria*	*Neisseria gonorrhoeae*	1.466	Non-contaminant
Proteobacteria	Betaproteobacteria	Burkholderiales	Burkholderiaceae	*Ralstonia*	*Ralstonia solanacearum*	1.514	Non-contaminant
Firmicutes	Bacilli	Bacillales	Bacillaceae	*Bacillus*	*Bacillus cereus*	1.538	Non-contaminant
Firmicutes	Clostridia	Clostridiales	Clostridiaceae	*Clostridium*	*Clostridium botulinum*	1.622	Non-contaminant
Proteobacteria	Gammaproteobacteria	Enterobacterales	Enterobacteriaceae	*Escherichia*	*Escherichia coli*	1.844	Non-contaminant
Proteobacteria	Gammaproteobacteria	Pseudomonadales	Pseudomonadaceae	*Pseudomonas*	*Pseudomonas putida*	2.249	Non-contaminant
Proteobacteria	Betaproteobacteria	Burkholderiales	Burkholderiaceae	*Burkholderia*	*Burkholderia pseudomallei*	3.421	Non-contaminant
Proteobacteria	Gammaproteobacteria	Pseudomonadales	Pseudomonadaceae	*Pseudomonas*	*Pseudomonas tolaasii*	5.701	Non-contaminant
Firmicutes	Bacilli	Bacillales	Staphylococcaceae	*Staphylococcus*	*Staphylococcus simulans*	6.015	Non-contaminant
Firmicutes	Bacilli	Bacillales	Staphylococcaceae	*Staphylococcus*	*Staphylococcus haemolyticus*	11.147	Non-contaminant
Firmicutes	Bacilli	Bacillales	Staphylococcaceae	*Staphylococcus*	*Staphylococcus aureus*	14.109	Non-contaminant
Proteobacteria	Gammaproteobacteria	Pasteurellales	Pasteurellaceae	*Pasteurella*	*Pasteurella multocida*	18.904	Non-contaminant
Proteobacteria	Gammaproteobacteria	Enterobacterales	Enterobacteriaceae	*Klebsiella*	*Klebsiella pneumoniae*	25.097	Non-contaminant
